# d-Cycloserine enhances the bidirectional range of NMDAR-dependent hippocampal synaptic plasticity

**DOI:** 10.1038/s41398-023-02725-7

**Published:** 2024-01-09

**Authors:** Stefan Vestring, Alexandra Dorner, Jonas Scholliers, Konstantin Ehrenberger, Andrea Kiss, Luis Arenz, Alice Theiss, Paul Rossner, Sibylle Frase, Catherine Du Vinage, Elisabeth Wendler, Tsvetan Serchov, Katharina Domschke, Josef Bischofberger, Claus Normann

**Affiliations:** 1 Department of Psychiatry and Psychotherapy, Medical Center – University of Freiburg, Faculty of Medicine, University of Freiburg, D-79104 Freiburg, Germany; 2 Berta-Ottenstein-Programme for Clinician Scientists, Faculty of Medicine, University of Freiburg, D-79110 Freiburg, Germany; 3 Centre National de la Recherche Scientifique (CNRS) UPR3212, Université de Strasbourg, Institut des Neurosciences Cellulaires et Intégratives (INCI), Strasbourg, France; 4 University of Strasbourg, Institute for Advanced Study (USIAS), Strasbourg, France; 5 Center for Basics in Neuromodulation (NeuoModulBasics), Faculty of Medicine, University of Freiburg, D-79106 Freiburg, Germany; 6 Department of Biomedicine, University of Basel, CH-4056 Basel, Switzerland

**Keywords:** Hippocampus, Depression

## Abstract

The partial N-methyl-D-aspartate receptor (NMDAR) agonist d-Cycloserine (DCS) has been evaluated for the treatment of a wide variety of psychiatric disorders, including dementia, schizophrenia, depression and for the augmentation of exposure-based psychotherapy. Most if not all of the potential psychiatric applications of DCS target an enhancement or restitution of cognitive functions, learning and memory. Their molecular correlate is long-term synaptic plasticity; and many forms of synaptic plasticity depend on the activation of NMDA receptors. Here, we comprehensively examined the modulation of different forms of synaptic plasticity in the hippocampus by DCS and its mechanism. We found that DCS positively modulates NMDAR-dependent forms of long-term synaptic plasticity (long-term synaptic potentiation, LTP, and long-term synaptic depression, LTD) in hippocampal brain slices of juvenile rats without affecting basal synaptic transmission. DCS binds to the d-serine/glycine binding site of the NMDAR. Pharmacological inhibition of this site prevented the induction of LTP, whereas agonism at the d-serine/glycine binding site augmented LTP and could functionally substitute for weak LTP induction paradigms. The most probable origin of endogenous d-serine are astrocytes, and its exocytosis is regulated by astrocytic metabotropic glutamate receptors (mGluR1). Functional eradication of astrocytes, inhibition of mGluR1 receptors and G-protein signaling in astrocytes adjacent to postsynaptic neurons prevented the induction of NMDAR-dependent forms of LTP and LTD. Our results support the enhancement of a bidirectional range of NMDAR-dependent hippocampal synaptic plasticity by DCS and d-serine-mediated gliotransmission. Therefore, the d-serine/glycine-binding site in NMDAR is a major target for psychopharmacological interventions targeting plasticity-related disorders.

## Introduction

The clinical development of d-Cycloserine (DCS) has experienced several waves. It was introduced in the 1960s as an antimicrobial agent for the treatment of tuberculosis. DCS was later found to be centrally active as a selective partial NMDAR agonist, acting at its serine/glycine-binding site (for review see [1]). Based on the assumption of glutamatergic deficits in dementia and schizophrenia, a number of clinical trials examined the treatment of Alzheimer disease [2] and negative symptoms of schizophrenia [3] in the 1990s. However, these treatment approaches largely failed [1, 4, 5]. A third wave was initiated by a seminal study of Ressler et al. in 2004, in which DCS successfully augmented virtual exposure therapy for height phobia [6]. In the following years, DCS has been used in many clinical trials to augment exposure-based cognitive behavior therapy for anxiety-related disorders, obsessive-compulsive disorder, addiction and posttraumatic stress disorder. However, a metaanalysis revealed only a small and potentially not clinically meaningful augmentation effect of DCS on exposure-based therapy [7]. More recently, first clinical trials support an antidepressant effect of DCS [8].

In animal models, many studies have found an amygdala-based facilitation of fear extinction and extinction retention and a reduction in reinstatement of learned fear after extinction by DCS [9]. Moreover, it has been demonstrated that hippocampus-dependent learning paradigms have also been facilitated by DCS in animals and humans [10–12].

These lines of evidence suggest that most if not all of the potential psychiatric applications of DCS might target an enhancement or restitution of learning and memory. The molecular correlate of learning and memory is long-term synaptic plasticity [13, 14]; and many forms of synaptic plasticity depend on the activation of NMDA receptors [15]. Therefore, the partial NMDAR agonist DCS is expected to modulate both synaptic plasticity and its behavioral output, learning and memory formation [16, 17]. Here, we comprehensively examined the modulation of different forms of synaptic plasticity in the hippocampus by DCS, its mechanism and the physiological significance of the involved signaling pathways.

## Materials and methods

### Animals and slice preparation

Juvenile Wistar rats (postnatal days 8–15) were used in all experiments. The main aim of the present study was to examine the modulation of prototypical forms of synaptic plasticity by DCS and its mechanisms; therefore, we decided to use juvenile animals where both LTP and LTD are readily inducible. Animal use and procedures were approved by the Regierungspräsidium Freiburg, Germany.

Transverse hippocampal brain slices were cut with a vibratome (300 µm, VT 1200, Leica Biosystems) ex vivo from the brains of rats sacrificed by decapitation in accordance with national and institutional guidelines. The slices were maintained at 34 °C for 25 min and then stored at room temperature in a saline solution containing (in mM): 125 NaCl, 2.5 KCl, 27 D(+)-Glucose, 2 CaCl_2_, 1 MgCl_2_, 1.25 NaH_2_PO_4_, and 25 NaHCO_3_ bubbled with carbogen (95% O_2_, 5% CO_2_).

### Electrophysiology

Picrotoxin (50 µM) was added to the saline solution to isolate excitatory neurotransmission. The slices were superfused in the recording chamber at a flow rate of 5–10 ml/min. CA1 pyramidal neurons were visually identified with infrared differential contrast video microscopy (Axioskop 2 FS plus, Zeiss; IMAGO-VGA, TILL Photonics). The whole-cell configuration was established using patch pipettes pulled from borosilicate glass tubing (outer diameter, 2 mm; wall thickness, 0.5 mm; and open pipette resistance, 3-5 MΩ); filled with an internal solution containing 135 K-gluconate, 20 KCl, 2 MgCl_2_, 2 Na2-ATP, 10 HEPES, 0.2 EGTA, 0.3 sodium guanosine 5′-triphosphate. For LTD measurements, the EGTA concentration was increased to 0.5, and the pH was equilibrated with KOH to 7.3. CA1 pyramidal cells were additionally identified by their characteristic adaptive firing frequency in response to long depolarizing current pulses. Their resting membrane potential was between −72 and −68 mV, and the holding potential was set to −70 mV. Series resistance Rs was monitored by interleaved short −5 mV current pulses every 100 sec. The average Rs was 21.8 ± 0.4 MΩ (min 7.6 MΩ, max. 44.4 MΩ, *n* = 609). These relatively high values for Rs were chosen to avoid excessive dialysis of the patched neuron while enabling current clamp measurements. Results were excluded when Rs exceeded 50 MΩ, no firing pattern could be induced at the beginning of the experiment, excessive noise was noticed, Rs and EPSP amplitudes changed symmetrically or Rs changed by <30% over the course of a measurement. Recordings were made with an EPC-10/2 double amplifier (HEKA) and analyzed with Pulse, Pulsefit (HEKA) and GraphPad Prism software. To evoke EPSPs, a stimulation pipette filled with HEPES-buffered NaCl solution was placed in the stratum radiatum of the CA1 region 20–50 µm away from the pyramidal cell layer. Voltage pulses (200 µs) of 10–100 V were applied at 0.1 Hz to reach an initial EPSP amplitude of 3–6 mV.

### Simultaneous pyramidal cell/astrocyte recordings

Brain slices were incubated directly after slicing for 20 min at 34 °C in saline with 0.5–1 µM sulforhodamine 101 (SR 101), an astrocyte-specific fluorescent dye. The supernatant was washed out for an additional 10 min in saline. The excitation light source (Polychrome II, TILL Photonics) was coupled with the epifluorescence port of the microscope via a light guide; the excitation wavelength was set to 578 nm, and the light intensity was reduced with a gray filter to 10% to minimize bleaching. Emission was detected with the IMAGO-VGA (TILL Photonics) fluorescence camera at 592 nm using a Zeiss filter combination. Adjacent CA1 pyramidal neurons and stratum radiatum astrocytes were visually identified. The astrocytes were characterized by their fluorescence, a membrane potential of approximately −85 mV, the absence of action potentials in response to depolarization, and the absence of EPSPs in response to Schaffer collateral stimulation. After establishing whole-cell access to astrocytes, GDP-ß-S (20 mM) was allowed to diffuse into cells and within the astrocytic network for 10–15 min. Thereafter, CA1 neurons were patched, a stable EPSP baseline was obtained, and LTP was induced.

### Chemicals

All chemicals were purchased from Sigma-Aldrich or Tocris, and stock solutions were prepared in distilled water, dimethyl sulfoxide, or NaOH, as appropriate. Most substances were applied by path perfusion with the exception of U73122, heparin, PKC19-36 and GDP-ß-S, which were added to the intracellular solution in the recording pipette and injected into the cell.

### Data analysis and statistics

**Fig. 1:**
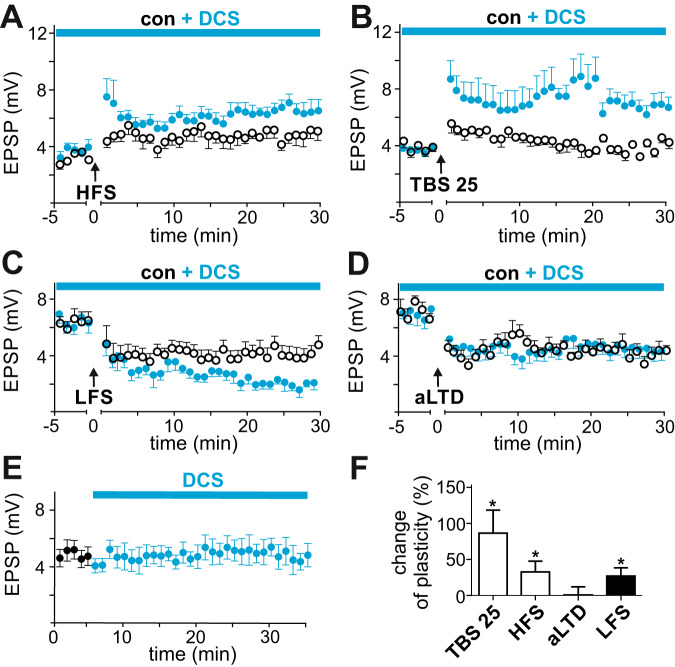
d-cycloserine (DCS) augments NMDA-dependent forms of synaptic plasticity. **A** Nonassociative LTP with two blocks of 100 Hz stimulation (HFS, black circles) caused significant LTP, which was augmented by DCS (blue dots). **B** A reduced associative form of LTP with only one theta block (TBS 25) led to no significant LTP. In the presence of 20 µM DCS, the TBS 25 protocol induced significant LTP. **C** Homosynaptic LTD was induced by low-frequency stimulation (LFS, 5 Hz for 10 min). LFS-LTD in control solution was augmented by DCS. **D** Associative LTD (aLTD) remained unchanged by bath application of DCS. **E** DCS had no effect on basal synaptic transmission. **F** Augmentation of the amount of different forms of synaptic plasticity by DCS (comparison in % of differences between baseline and EPSP amplitudes 20–30 min after induction. White bars, LTP; black bars, LTD). Asterisks indicate significant differences between the amount of plasticity in the control solution and in the presence of DCS.

**Fig. 2:**
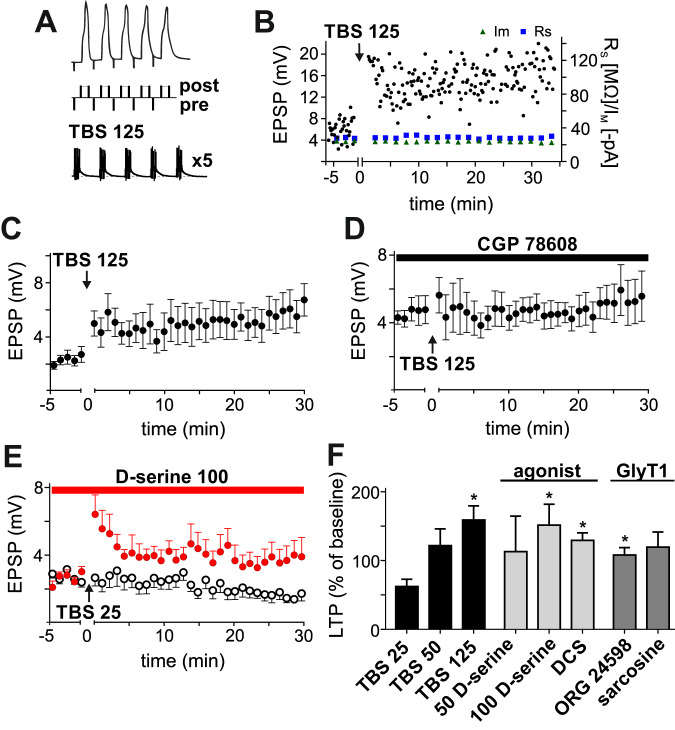
Pharmacological activation of the d-serine/glycine NMDR binding site augments NMDAR-dependent forms of LTP. **A** Associative LTP induction protocol. EPSPs were evoked by Schaffer collateral stimulation and synchronized with postsynaptic electrically evoked action potentials in CA1 pyramidal cells. Five AP/EPSP pairings were applied at 100 Hz; this burst was repeated five times at 5 Hz, and the resulting theta block was generated five times with an interval of 10 sec, resulting in a total of 125 AP/EPSP pairings. **B** Representative single experiment. Black dots, maximal EPSP amplitudes before and after LTP induction with the theta-burst pairing protocol (TBS 125); blue squares, series resistance; green triangles, membrane current evoked by a hyperpolarizing pulse of −5 mV. **C** The TBS 125 protocol caused a stable induction of LTP in the control solution. **D** In the presence of CGP 78608 (100 nM), an antagonist of the d-serine/glycine binding site in NMDAR, LTP induction was inhibited. **E** Available glutamate during LTP induction was decreased by reducing the number of EPSPs to one in the theta burst protocol, resulting in 25 AP/EPSP pairings (TBS 25). This protocol did not induce a significant LTP. Bath application of d-serine (100 µM) rescued LTP. **F** Amount of LTP under different experimental conditions. Asterisks mark significant differences compared to that induced in the TBS 25 protocol. One (TBS 25) and two (TBS 50) EPSPs during the theta-burst protocol did not induce significant LTP compared to that induced by 5 EPSPs (TBS 125). d-serine at 100 µM but not d-serine at 50 µM, and d-cylocloserine (DCS, 20 µM) significantly enhanced TBS 25. The glycine reuptake inhibitor (GlyT1) ORG 24598 (10 µM), but not sarcosine (30 µM), significantly augmented TBS 25.

**Fig. 3:**
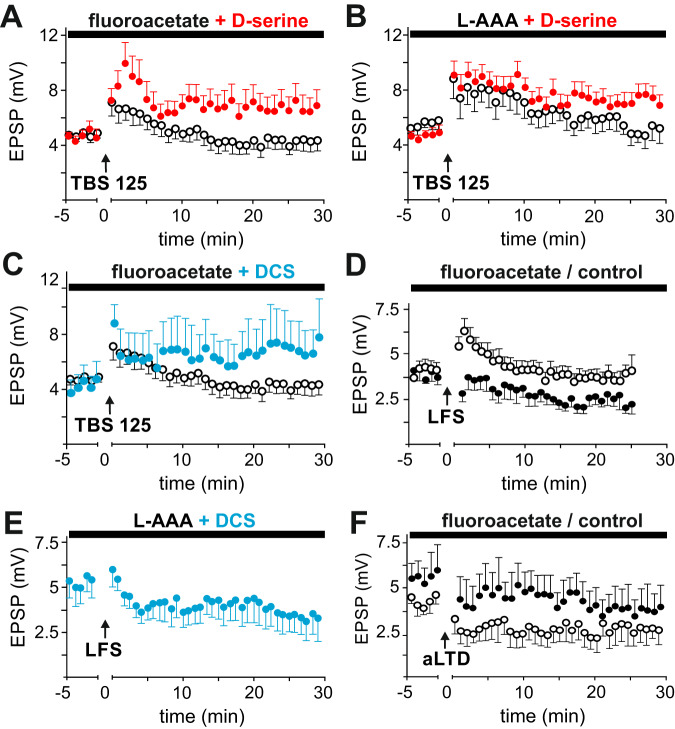
Modulation of synaptic plasticity by astrocyte function. **A** Functional astrocytes were eliminated by preincubation of brain slices with the glial cell-specific metabolic inhibitor sodium fluoroacetate (3 mM, black circles). Bath application of d-serine (100 µM) after preincubation with fluoroacetate rescued LTP induction (red dots). **B** Functional astrocytes were eliminated by preincubation of brain slices with the excitotoxin (S)-2-aminohexanedioic acid (L-AAA, 1 mM), which blocked LTP induction (black circles). Again, bath application of d-serine (100 µM) rescued LTP induction (red dots). **C** The addition of DCS (20 µM, blue) restored LTP in slices preincubated with 3 mM fluoroacetate (open circles). **D** An NMDA-dependent form of LTD was induced by low-frequency stimulation (LFS) of Schaffer collaterals at 5 Hz for 10 min and resulted in a significant decrease in EPSP amplitudes (black dots, separate series as in Fig. 1C). LFS-LTD induction was inhibited after preincubation with fluoroacetate (3 mM; open circles). **E** After preincubation with L-AAA and in the presence of DCS, a significant LFS-LTD could be induced. **F** NMDA-independent aLTD caused a stable reduction in EPSP amplitudes (black dots, different experimental series than Fig. 1D). After elimination of functional astrocytes by preincubation with fluoroacetate (3 mM), the LTD level was unchanged compared to that measured under control conditions (open circles). Note the different baseline EPSP amplitudes.

**Fig. 4:**
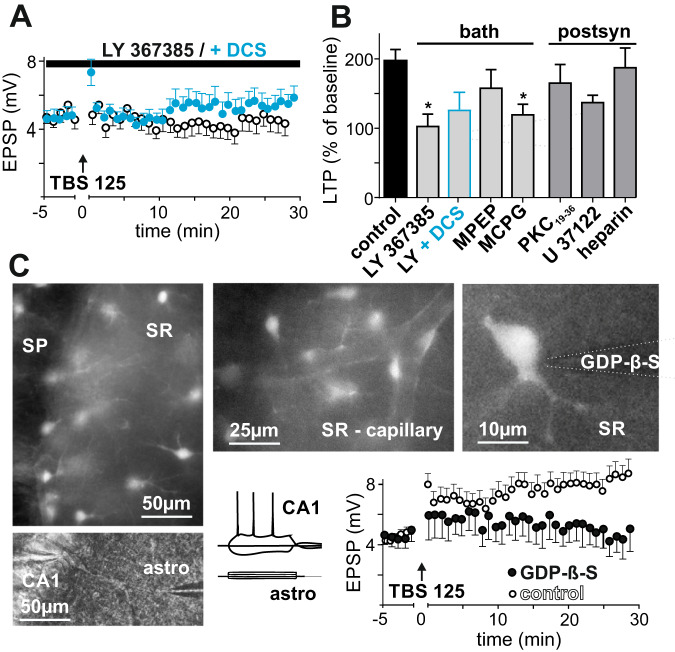
Role of astrocytic G-protein signaling and mGluR1 activation for LTP. **A** The TBS 125 protocol was applied in the presence of the selective mGluR1a antagonist LY 367385 (100 µM) in bath solution, which inhibited LTP (black circles). Control TBS 125 LTP as in Fig. 3A. DCS (20 µM) partially restored LTP induction in the presence of LY 367385 (blue dots). **B**. LTP in response to the TBS 125 induction protocol (% of averaged EPSP amplitudes 20–30 min after induction vs. 0–5 min before induction, control LTP depicted in Fig. 3A). Asterisks indicate significant differences from control LTP. LTP was significantly inhibited by bath application of LY 367385, but partially restored by DCS (20 µM) in the presence of LY 367385. The nonselective group I/II mGluR antagonist (S)-MCPG (500 µM) but not by the mGluR5 antagonist MPEP (10 µM) blocked Induction of LTP. Infusion of PKC/PLC pathway inhibitors in the postsynaptic pyramidal neuron via patch pipette did not alter LTP (PKC19-36, 10 µM; U 73122, 20 µM; heparin, 4 mg/ml). **C** Hippocampal brain slices were incubated with the astrocyte-specific fluorescent dye sulforhodamine 101, and simultaneous whole-cell recordings of astrocytes and nearby pyramidal cells were established. Upper left panel: marked astrocytes in CA1 (SP, stratum pyramidale and SR, stratum radiatum). Upper middle panel: Astrocytes accumulated near capillaries. Lower left panel: localization of astrocyte and pyramidal cell measurements in CA1, representative image. Right panel: GDP-β-S was infused through patch pipette (shape enhanced) in astrocytes in the stratum radiatum. Lower right panel: response of pyramidal cells and astrocytes to long depolarizing pulses. **I**ntracellular application of GDP-β-S (20 mM) to astrocytes significantly inhibited LTP at nearby CA3-CA1 synapses (black dots). Control condition (black circles): nearby astrocytes were patched without GDP-β-S in the intracellular solution; a significant LTP could be induced.

**Fig. 5:**
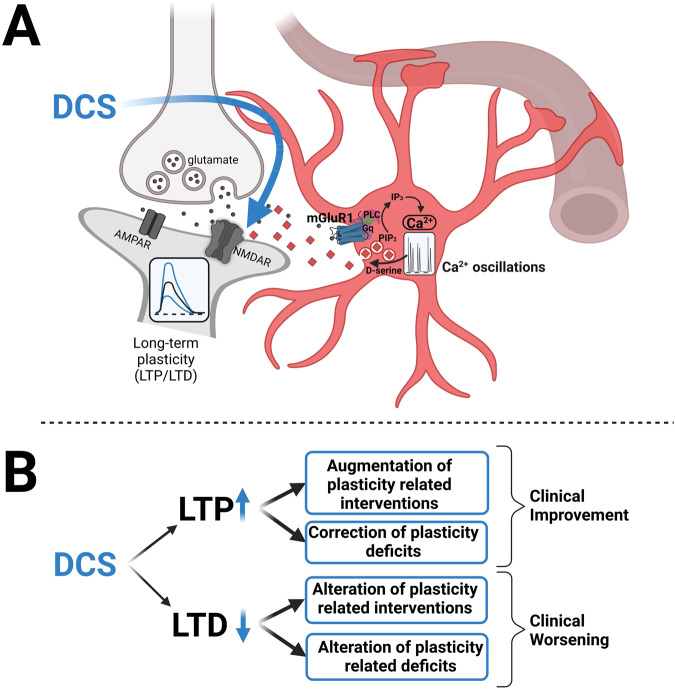
Schematic overview of the modulation of synaptic plasticity by DCS. **A** Spill-over of glutamate from presynaptic pyramidal cells activates mGluR1 at nearby astrocytes. Activation of astrocytic mGluR1 leads to PLC activation, converting PIP_2_ to IP_3_ and DAG, and leads to specific calcium oscillations that trigger d-serine release from astrocytes. d-serine acts as coactivator at postsynaptic NMDARs and augments NMDAR-dependent forms of long-term synaptic plasticity. Exogenous DCS directly binds to the d-serine/glycine binding site at NMDARs and enhances NMDAR currents. **B** Hypothetical functional consequences of the bidirectional augmentation of NMDA-dependent synaptic plasticity by DCS: DCS increases the amplitude of long-term potentiation (LTP) and long-term depression (LTD), which could result either in clinical improvement or worsening. For details, see “Discussion”.

Since no valid information on expected effect sizes were available, no power calculation was performed. Every experimental condition contained a minimum of five recordings from different animals. Randomization was performed between experimental series and the corresponding control condition using https://www.randomizer.org/ software to determine whether experimental or the control experiment was performed. The experimenter was not blinded to the experimental condition. All values are given as the means ± SEM. In amplitude-time plots, the average EPSP amplitudes were calculated from the means of five to seven consecutive EPSPs; error bars represent SEM. Within series, the previous 60 EPSPs before exposure to a substance or plasticity induction were compared to 100 EPSPs obtained 20–30 min after termination of the intervention. For normal distribution, two-tailed paired t-tests were used for within-group comparisons and unpaired t-tests for between-group comparisons. Normal distribution was tested using Shapiro Wilk normality test. If test results did not meet criteria for normal distribution, Mann-Whitney or Wilcoxon matched-pairs rank tests were used with a significance level of 0.05. Statistics were analyzed and figures were made with GraphPad Prism software (Figs. 1–4) and Biorender.com (Fig. 5).

## Results

### d-Cycloserine augments NMDAR-dependent forms of synaptic plasticity in the hippocampus

DCS binds to the d-serine/glycine binding site which is located on the NR1 subunit in NMDAR [18], increasing its open state probability and time [19]. d-serine is the dominant endogenous ligand of the binding site [20]. This binding is necessary for the opening of the NMDAR channel pore [21, 22]; and [Ca^2+^] influx through postsynaptic NMDARs is a necessary initial signal for the induction of many forms of long-term synaptic plasticity [15].

We examined the modulation of different forms of synaptic plasticity by DCS in hippocampal brain slices obtained from juvenile rats (postnatal days 8-15). In all experiments, we used a concentration of 20 µM DCS in the bath solution which should correspond to therapeutic brain concentrations in humans [23].

First, a non-associative form of long-term synaptic potentiation (LTP) was induced by tetanic high-frequency stimulation (HFS) of the Schaffer collaterals at 100 Hz for 2 × 1 s with an interval of 2 s. The canonical 4 × 1 s stimulation protocol was not used to prevent ceiling effects. HFS in control solution induced significant LTP, to 150.1 ± 12.2% of the baseline (*n* = 8, *p* = 0.0078 vs. the baseline); the addition of DCS to the bath solution significantly augmented LTP, to 183.1 ± 9.0% (*n* = 8, *p* = 0.0078 vs. the baseline, *p* = 0.0464 vs. HFS; Fig. 1A). Then, we tested a low-intensity associative theta burst stimulation protocol. Five excitatory postsynaptic potentials (EPSPs) induced by Schaffer collateral stimulation at 100 Hz were paired with action potentials (APs) induced by short depolarization of CA1 pyramidal neurons. These five pairings were repeated five times at a frequency of 20 Hz, resulting in 25 pairings (TBS 25, 106.4 ± 6.7% of the baseline EPSP amplitudes, *n* = 7, *p* = 0.5781 vs. the baseline). Bath application of DCS with the TBS 25 protocol resulted in significant LTP to 194.1 ± 25.3% (*n* = 11, *p* = 0.0010 vs. the baseline EPSP amplitudes, *p* = 0.0164 vs. TBS 25; Fig. 1B). These results support the potential of DCS to effectively augment hippocampal LTP.

Whereas virtually all forms of LTP are NMDAR-dependent, the postsynaptic target of glutamate in long-term synaptic depression (LTD) induction can differ: Homosynaptic forms of LTD are NMDAR-dependent; associative spike time-dependent forms of LTD are NMDA-independent and require the activation of postsynaptic metabotropic glutamate receptors (mGluR) together with high-voltage activated Ca^2+^ channels [24]. Homosynaptic LTD was induced by subthreshold low frequency stimulation (LFS) of the Schaffer collaterals, 5 Hz, for 10 min (67.5 ± 10.1% of baseline, *p* = 0.0234 vs. the baseline, *n* = 8). DCS significantly increased LFS-LTD, to 40.8 ± 4.6% (p = 0.0005 vs. the baseline, *p* = 0.0383 vs. LFS, *n* = 7; Fig. 1C). Associative LTD (aLTD) was induced by asynchronous AP-EPSP pairings and resulted in stable LTD compared to the baseline EPSP amplitudes (65.0 ± 9.2%, *p* = 0.0313 vs. the baseline, *n* = 7). DCS did not alter the amount of LTD (64.1 ± 7.0%, *n* = 7, *p* = 0.0156 vs. the baseline, *p* = 0.9408 vs. aLTD; Fig. 2C). In wash-in experiments, DCS had no effect on basal synaptic transmission (4.8 ± 0.7 mV before wash-in, 4.8 ± 0.7 mV 25–35 min after wash-in, *n* = 8, *p* = 0.5234, Fig. 1E). Taken together, the findings show that DCS selectively augments all tested forms of NMDAR-dependent synaptic plasticity (NMDA-dependent forms of plasticity: TBS 25, LTP was augmented by DCS by 87.6 ± 32.5% compared to the control solution, p = 0.0164; at HFS, 33.0 ± 15%, *p* = 0.0464; at LFS, 26.8 ± 11.6%, p = 0.0383; and NMDA-independent form, aLTD 0.9 ± 11.6%, *p* = 0.9408; Fig. 1F).

### Hippocampal LTP is bidirectionally modulated by the NMDAR d-serine/glycine binding site

We then examined the modulation of hippocampal LTP by pharmacological manipulation of the d-serine/glycine binding site. Associative LTP was induced by theta-burst stimulation (TBS 125). The EPSP-AP pairings used in Fig. 1D were repeated five times with an interval of 10 sec, resulting in a total of 125 EPSP-AP pairings (Fig. 2A). This protocol resulted in a stable increase in the EPSP amplitude, to 183.4 ± 28.6% of the baseline (p = 0.0042, *n* = 9, Fig. 2B, C). CGP 78608 selectively inhibits the d-serine/glycine binding site of the NMDAR [25]. In the presence of 100 nM CGP 78608, no significant LTP was induced by the associative induction protocol (107.6 ± 20.3% of the baseline, *p* = 0.6875, *n* = 7; *p* = 0.0421 vs. control LTP; Fig. 2D).

Furthermore, we tested whether different means of pharmacological activation of the d-serine/glycine binding site in NMDAR can augment LTP. To avoid ceiling effects, we reduced the strength of the LTP induction paradigm and therefore the amount of postsynaptically available glutamate by decreasing the number of EPSPs in the TBS-LTP protocol. When only the first AP in every theta-burst block was paired with an EPSP (resulting in 25 EPSP-AP pairings, TBS 25), the average baseline EPSP amplitude was reduced to 62.7 ± 10.7%, but did not reach a level of significance (*p* = 0.0648, *n* = 8; Fig. 2E). Pairing of the first and third AP with an EPSP increased the EPSP amplitude to 122.9 ± 24.9% of the baseline (TBS 50, *p* = 0.3750, *n* = 7, *p* = 0.0721 vs. TBS 25; Fig. 2F), whereas all 125 AP-EPSP pairings produced significant LTP, to 160.9 ± 20.8% of the baseline (TBS 125, *p* = 0.0234, *n* = 8, *p* = 0.0011 vs. TBS 25; Fig. 2F) in this set of experiments. Bath application of 100 µM d-serine significantly augmented the TBS 25 induction protocol (153.2 ± 31.0% of the baseline EPSP amplitude, *p* = 0.2031, *n* = 9, *p* = 0.0464 vs TBS 25; Fig. 2E). Moreover, DCS (20 µM, 130.7 ± 11.0%, *p* = 0.0061, *n* = 13, *p* = 0.0002 vs. TBS25) and the selective glial glycine transporter GlyT1b antagonist ORG 24598 (10 µM, 109.2 ± 10.9%, *p* = 0.6250, *n* = 10, *p* = 0.0117 vs. TBS25) significantly augmented synaptic potentiation. A lower concentration of d-serine (50 µM, 114.5 ± 52.7%, *p* = 0.9999, *n* = 5, *p* = 0.7242 vs. TBS 25) and the GlyT1 inhibitor sarcosine (30 µM, 120.9 ± 22.1%, *p* = 0.2500, *n* = 9, *p* = 0.1288 vs. TBS 25) numerically increased potentiation but not to a significant level compared to that of the control solution (Fig. 2F). Taken together, we found a bidirectional modulation of LTP depending on the activation of the d-serine/glycine binding site of the NMDAR: pharmacological inhibition of the serine/glycine binding site prevented LTP induction, whereas increased binding at this site by direct agonism or by glycine reuptake inhibition augmented LTP.

### Modulation of synaptic plasticity by astrocyte function

So far, our results suggest a potent regulation of synaptic plasticity by pharmacological modulation of the NMDAR d-serine/glycine binding site. If this were the case, another promising target to interact with synaptic plasticity would be the regulation of the availability of its endogenous ligand, d-serine. d-serine is synthesized in glial cells and postsynaptic neurons by serine racemase [22, 26–28]. In the following set of experiments, we focused on the gliotransmission hypothesis, stating that d-serine is released by exocytosis from hippocampal astrocytes after the binding of glutamate to astrocytic mGluRs [29, 30].

First, functional astrocytes were eliminated by preincubation of the brain slices with sodium fluoroacetate (3 mM) or (S)-2-aminohexanedioic acid (L-AAA, 1 mM). Fluoroacetate is an astrocyte-specific blocker of cellular metabolism that inhibits glial aconitase [31], whereas L-AAA enters cells via Na^2+^-dependent transporters and specifically induces glial cell death [32]. Fluoroacetate blocked spike time-dependent LTP induction to 90.9 ± 14.0% of its baseline (*p* = 0.6257, *n* = 14; *p* = 0.0030 vs. control LTP; Fig. 3A). A significant level of LTP was rescued by the addition of 100 µM d-serine in the bath solution (158.7 ± 25.4% of the baseline, *p* < 0.0426, n = 12; *p* = 0.2139 vs. control LTP; Fig. 3A). The addition of 10 µM d-serine numerically increased synaptic strength, but this change did not reach the level of significance (120.2 ± 19.5% of the baseline, *p* > 0.5570, n = 10; *p* = 0.0223 vs. control LTP; data not shown). In the presence of L-AAA, no significant LTP was induced (104.7 ± 13.6% of the baseline, *p* = 0.6518, *n* = 9; *p* = 0.0010 vs. control LTP; Fig. 3B). d-serine (100 µM) restored the LTP level (164.6 ± 15.7% of the baseline, *p* < 0.0124, *n* = 12; *p* = 0.5458 vs. control LTP; Fig. 3B). After preincubation with fluoroacetate, DCS rescued LTP (158.9 ± 19.6% of the baseline, *p* < 0.0472, *n* = 5; *p* = 0.5736 vs. control LTP, Fig. 3C).

We also tested the effect of the functional eradication of astrocytes on different forms of hippocampal LTD. First, homosynaptic NMDA-dependent LTD was induced by prolonged low-frequency stimulation of Schaffer collaterals (LFS). Stimulation with 5 Hz for 10 min resulted in significant LTD, to 64.7 ± 3.4% of the baseline (*p* = 0.0001, *n* = 7). Preincubation with fluoroacetate prevented LTD induction (88.5 ± 4.8% of the baseline, *n* = 10, *p* = 0.0520; *p* = 0.0022 vs. control LTD; Fig. 3D). After preincubation with L-AAA and in the presence of DCS, a significant LTD could be induced (82.63 ± 7.2% of the baseline, *n* = 6, *p* < 0.0349; *p* = 0.5578 vs. control LFS-LTD; Fig. 3E). The NMDA-independent aLTD protocol led in a separate set of experiments to stable depression of EPSP amplitudes, to 77.4 ± 14.3% of baseline (*p* = 0.0156, *n* = 7). After preincubation with fluoroacetate, the amount of aLTD was unchanged (70.4 ± 15.8% of the baseline, *p* = 0.0391, *n* = 8; *p* = 0.6126 vs. control LTD, Fig. 3F). These results suggest that the presence of functional astrocytes or exogenous d-serine are necessary for the induction of NMDA-dependent forms of synaptic plasticity.

### Role of astrocytic G-protein signaling and mGluR1 activation for LTP

In a next step, we examined the role of astrocytic mGluR and G-protein signaling for LTP induction. TBS 125 caused significant LTP in this set of experiments (197.2 ± 16.0% of the baseline, *p* = 0.0001, *n* = 35, Fig. 4B). First, mGluR antagonists were added to the bath solution. The selective mGluR1a antagonist LY 367385 (100 µM) strongly inhibited LTP (102.7 ± 17.9% of the baseline, p = 0.7646. *n* = 11; *p* = 0.0003 vs. control LTP). Combined bath application of LY 367385 and DCS partially restored LTP (128.8 ± 8.8% of the baseline EPSP amplitudes, *n* = 8, *p* = <0.0097; *p* = 0.2073 vs. control LTP; Fig. 4A, B). The selective, noncompetitive mGluR5 antagonist MPEP (10 µM) had no significant effect on LTP compared to the control experiments; however, no significant LTP was induced in the presence of MPEP (157.7 ± 26.7% of the baseline, *p* = 0.1563, n = 7; *p* = 0.2801 vs. control LTP, Fig. 4B). (S)-MCPG (500 µM) is a nonselective group I/II mGluR antagonist, and treatment with (S)-MCPG resulted in LTP inhibition (119.2 ± 15.2% of the baseline, p = 0.1868, n = 5; p = 0.0190 vs. control LTP, Fig. 4B).

These results demonstrate that LTP depends on the activation of mGluR1a receptors. However, application of antagonists in the bath solution did not allow us to locate their target cells. We therefore applied inhibitors of the PKC/PLC pathway intracellularly via patch pipette into postsynaptic CA1 neurons to block signal transduction downstream of postsynaptic mGluR. Neither the specific PKC inhibitor PKC19-36 (10 µM, 165.2 ± 26.7% of the baseline, *p* = 0.0210, *n* = 12; *p* = 0.1918 vs. control LTP), the selective PLC inhibitor U 73122 (20 µM, 137.0 ± 10.7% of the baseline, *p* = 0.0156, *n* = 8; *p* = 0.0727 vs. control LTP), nor the IP_3_ receptor antagonist heparin (4 mg/ml, 187.3 ± 28.5% of the baseline, *p* = 0.0078, *n* = 8; *p* = 0.8149 vs. control LTP; Fig. 4B) significantly inhibited LTP. These results exclude a role for postsynaptic mGluR in LTP.

To assess directly the role of astrocytic G-protein signaling, we used a combined fluorescence-double-patch method. Acute hippocampal brain slices were incubated with the astrocyte-specific fluorescent dye sulforhodamine 101. A visually identified fluorescent astrocyte located in the stratum radiatum adjacent to the apical dendrite of the target CA1 pyramidal neuron was then patched and electrically identified by the absence of action potential in response to depolarization, no EPSPs and a membrane potential of approximately −85 mV. We then allowed GDP-β-S (20 mM) to diffuse into astrocytes for 10–15 min through an open patch pipette and via gap junctions through the astrocytic network. GDP-β-S inactivates signaling pathways downstream of G protein-coupled receptors and was chosen because of its intracellular mechanism of action and its relatively low molecular weight, allowing the substance to spread through gap junctions. In previous work, infusion of GDP-β-S in adult astrocytes suppressed both expanded and focal [Ca^2+^]_i_ activity in astrocytic processes [33]. We patched a nearby CA1 neuron, and LTP was induced as described previously. Under these conditions, LTP induction was inhibited (118.7 ± 15.9% of the baseline, *p* **≥** 0.3125, *n* = 5; *p* = 0.0370 vs. control LTP, Fig. 4C). Taken together, these results suggest that both astrocytic G-protein signaling and activation of mGluR1 are necessary for LTP induction, most probably due to astrocytic mGluR1-regulated serine/glycine exocytosis.

## Discussion

### Exogenous activation of the NMDAR d-serine/glycine binding site augment NMDAR-dependent forms of synaptic plasticity

Few studies so far have explicitly examined the effect of DCS on hippocampal synaptic plasticity with inconsistent results. Rouaud and coworkers have found an increase in homosynaptic NMDAR-dependent forms of LTP and LTD; together with a decrease in AMPA-mediated synaptic transmission [16]. Zhang et al. described an increase of LTP only by a very low dose of DCS (1 µM), whereas higher doses (10 and 100 µM) inhibited LTP. 100, but not 10 µM DCS increased LTD [34]. Bath application of 50 or 100 µM DCS had no significant effect on LTD, but enhanced baseline synaptic transmission in another set of experiments [35]. In previously performed field potential experiments, NMDA-dependent LTD and spatial memory were positively modulated by both exogenous and astrocyte-derived d-serine [36]. In healthy humans, double-blinded single administration of 100 mg DCS enhanced the amplitude of motor-evoked potentials after 10 Hz repetitive transcranial magnetic stimulation (rTMS), a paradigm used to elicit a correlate of LTP in the motor cortex [17].

In our study, we found that an activation of the serine/glycine binding site of the NMDA receptor by DCS, d-serine and a GlyT inhibitor augmented NMDA-dependent forms of hippocampal long-term synaptic plasticity, whereas a selective antagonist of this binding site inhibited LTP. Pharmacological activation of the d-serine/glycine binding site could functionally substitute for weak induction paradigms and thereby decreased amounts of postsynaptically available glutamate. Basal AMPA-mediated synaptic transmission remained unchanged by DCS.

### Endogenous d-serine from astrocytes modulates synaptic plasticity

From our experiments, we conclude that astrocytes are the most probable origin of endogenous d-serine and that its exocytosis is regulated by astrocytic mGluR1 receptors. Since the 2000s, the concepts of gliotransmission and tripartite synapses have emerged in reference to the control of synapse formation and function [21, 37]. Different types of astrocyte plastically control distinct brain territories that might comprise thousands of synapses that are variably interconnected by gap junctions [38, 39]. The excitation of astrocytes is chemically induced and occurs through spillover of synapse-released neurotransmitters, which bind to astrocytic receptors and generate [Ca^2+^]_i_ oscillations [40, 41]. In response to excitation, astrocytes release gliotransmitters by exocytosis [42] (Fig. 5A).

A prominent example of gliotransmission in the CA1 region of the hippocampus is the release of d-serine after the binding of glutamate to astrocytic mGluR [29]. d-serine is synthesized in glial cells, but also in postsynaptic neurons, by serine racemase [22, 26, 27]. It is present in significant amounts in many areas of the brain, including the hippocampus, with a distribution similar to that of NMDARs [43]. SNARE-dependent exocytosis is triggered by [Ca^2+^]_i_ transients, which originate from IP_3_-sensitive intracellular Ca^2+^ stores and from the activation of kainate and metabotropic glutamate receptors [44].

Given the major importance of postsynaptic [Ca^2+^] influx through NMDAR for the induction of many forms of long-term synaptic plasticity, a modulating role for gliotransmission in LTP and LTD seems obvious [45]. In 2010, however, two nearly simultaneous high-impact publications presented contradictory results regarding the modulation of long-term synaptic plasticity by gliotransmission. Henneberger et al. [46] clamped [Ca^2+^]_i_ in astrocytes by intracellular application of the Ca^2+^ buffer EGTA and a fixed [Ca^2+^]_i_ concentration. Under these conditions, LTP at nearby Schaffer collateral-CA1 synapses was blocked and was restored by d-serine. In contrast, Agulhon et al. [47] used an IP_3_R2 knockout mouse model in which G_q_ protein-coupled receptor signaling in astrocytes was obliterated and found no modification of synaptic transmission or plasticity despite the absence of astrocytic [Ca^2+^]_i_ signaling. A few years later, Sun et al. [48], suggested that gliotransmission, at least gliotransmission mediated by astrocytic mGluR5 signaling, was virtually absent in adulthood.

These seemingly conflicting results have stimulated a fierce and ongoing debate on the existence of gliotransmission per se and especially the putative modulation of synaptic plasticity by astrocyte-derived gliotransmitters [49–51]. In any case, highly developmental, spatial and temporal variability in gliotransmission has become obvious, as has the heterogeneous impact on distinct forms of synaptic transmission and plasticity. Our results together with ample evidence from the literature strongly support a decisive role for d-serine-mediated gliotransmission in hippocampal synaptic plasticity [46].

Other groups have examined the modulation of d-serine-mediated gliotransmission on plasticity-related behavior. In these studies, functional glial ablation by fluoroacetate resulted in impairment to spatial learning and memory with impaired hippocampal LTP, all of which could be reversed by treatment with exogenous d-serine [52]. Impaired LTP and memory in aging mice and in GFAP-CB_1_-KO mice was shown to be rescued by exogenous d-serine [53].

Another fierce controversy in the field of gliotransmission concerns the origin of endogenous d-serine. Some groups argue that serine racemase is preferentially expressed in neurons [54]. In this model, astrocytes deliver l-serine via a serine shuttle [55] to neurons which than convert l-serine to D-serine by the SR [56]. From there, d-serine is not supposed to be released by Ca^2+^-dependent exocytosis but tonically by the neuronal alanine-serine-cystein transporter 1 (Asc-1) [57]. Advocates of this model do not question a role for d-serine in the regulation of synaptic plasticity, but they argue that D-serine is rather a co-transmitter or an autocrine substance than a gliotransmitter [28, 58]. In the case of traumatic brain injury or inflammation, astrocytic d-serine might gain a more prominent role and neuronal SR activity is downregulated [59]. A predominant neuronal origin of d-serine has been severely questioned; in the light of a possible role of neuronal SR for degradation of d-serine and for Asc-1 in its neuronal uptake, not efflux; together with the lack of a regulated d-serine release machinery in neurons [27, 60]. Many of our finding are compatible with both models; however, our results support a decisive role of group I mGluR-regulated exocytosis from astrocytes for LTP induction.

### Regulation of d-serine-mediated gliotransmission by astrocytic mGluR1a

Group I mGluR activates G_q_ and PLC, which results in the generation of [Ca^2+^]_i_ signaling after IP_3_-mediated release from intracellular stores. At least three mechanistic options may explain how mGluRs are involved in the modulation of synaptic plasticity by astrocytes: (a) mGluRs on astrocytes may elicit [Ca^2+^]_i_ oscillations that cause exocytosis of d-serine or glutamate as gliotransmitters. In turn, glutamate binds to (b) presynaptic or (c) postsynaptic mGluRs on neurons.

mGluR1 or truncated splice variants have been expressed in cultured rat hippocampal astrocytes [61, 62]. Hippocampal astrocytic [Ca^2+^]_i_ transients were prevented by treatment with the nonselective group I/II mGluR antagonist MCPG [63] or by the mGluR5 antagonist MPEP [64]. In other cortical regions, the combined application of mGluR1 and mGluR5 antagonists were previously shown to inhibit astrocytic [Ca^2+^]_i_ signaling [30]. As demonstrated in our experiments, LTP depends on mGluR activation and is selectively blocked by inhibition of G-protein-related signal transduction pathways in astrocytes by GDP-β-S infusion. Together with the previous literature, these findings are compatible with a decisive role for astrocytic mGluR receptors in gliotransmission and LTP induction. However, our data support a more prominent role of the mGluR1a subtype than indicated by previous results.

Our mGluR-related experiments cannot exclude a role for presynaptic group I mGluR, which may be activated by glutamate exocytosis from astrocytes. As shown in previous studies, [Ca^2+^]_i_- and SNARE-dependent glutamate release from astrocytes potentiated transmitter release at CA3-CA1 synapses by binding to presynaptic group I mGluRs, notably the mGluR1, and induced an NMDA-independent form of LTP by coincidental postsynaptic depolarization [65]. Similar mechanisms have been shown for spontaneous glutamate released from astrocytes, which binds to presynaptic group I mGluR and can regulate the threshold for associative LTP [66]. However, we consistently found a major role for the gliotransmitter d-serine in the astrocyte-dependent modulation of LTP, which makes the binding of astrocyte-derived glutamate at presynaptic mGluR unlikely to be functionally relevant.

In a prior publication [24], we showed that associative LTD depends on the activation of postsynaptic group I mGluR. Here, we excluded a role for postsynaptic mGluR in LTP induction due to the lack of intracellular effects of applied inhibitors of the PLC/PKC pathway in CA1 pyramidal neurons.

### Implications for psychopharmacology

Our results suggest that DCS and, more generally, the modulation of gliotransmission, could be a promising target for treatment modalities in psychopharmacology that depend on the augmentation of plasticity. However, the clinical outcome of the use of DCS in psychopharmacology has been inconsistent.

In order to reconcile conflicting clinical results, we hypothesize that in neurodegenerative disorders as Alzheimer and negative symptoms of schizophrenia, the potential of the brain to undergo plastic changes is severely reduced so that DCS has no relevant effect. Second, a continuous application of DCS has a risk for rapid tachyphylaxis [67]. Third, the most promising effects of DCS might be expected when combining its application with learning-dependent behavioral interventions; but the bidirectional augmentation of both LTP and LTD has to be considered (Fig. 5B).

The use of DCS in the treatment of major depressive disorder (MDD) could address two basic mechanisms. First, DCS could be used to augment adaptive learning in MDD by psychotherapeutic interventions [68]. Few clinical studies are currently examining an augmentation of cognitive behavioral, non-exposure based psychotherapies by DCS, but definite results are not yet available [69]. However, the bidirectional modulation of plasticity by DCS might also attenuate learning in psychotherapy. Second, many findings from animal models of depression and depressed humans suggest a dysregulation of cortical and hippocampal synaptic plasticity. Antidepressant interventions, including SSRIs, ketamine and non-invasive brain stimulation positively modulate synaptic plasticity (for review see [70, 71]). First clinical trials support an antidepressant effect of high, but not low doses of DCS in the monomodal treatment of MDD [8, 72, 73]. However, in animal models of depression, stress-facilitated LTD is normalized by antidepressant interventions [74], whereas DCS would further augment LTD. Further studies are necessary to clarify a potential benefit-risk ratio of DCS or other gliotransmission-related substances in MDD and other psychiatric disorders.

## Data Availability

The data that support the findings of this study are available from the corresponding author upon reasonable request.

## Material availability

The materials that support the findings of this study are available from the corresponding author upon reasonable request.
